# A new clinical trial to test high-dose vitamin C in patients with COVID-19

**DOI:** 10.1186/s13054-020-02851-4

**Published:** 2020-04-07

**Authors:** Anitra C. Carr

**Affiliations:** Department of Pathology & Biomedical Science, University of Otago, Christchurch, PO Box 4345, Christchurch, 8140 New Zealand

With the 2019 novel coronavirus (2019-nCoV) outbreak now spreading across the world, people are seeking ways in which to potentially protect themselves from the virus or to alleviate its effects once caught. One such means that is being touted online and in the media is vitamin C.

Vitamin C is best known for its antioxidant properties, being able to scavenge damaging reactive oxygen species, thus protecting the body’s cells and tissues from oxidative damage and dysfunction. However, the vitamin also has numerous other important functions within the body, many of which are known to support healthy immune function. During infection, vitamin C levels can become depleted and a person’s requirement for vitamin C increases with the severity of the infection [1]. In severe cases, this may require intravenous administration of gram doses in order to achieve high enough levels in the body to compensate for the enhanced turnover of the vitamin.

As of February 2020, the clinical characteristics of patients hospitalized with COVID-19-related pneumonia indicated that 26% were transferred to the ICU because of complications such as ARDS and shock [2]. A recently published RCT carried out in the USA in 167 patients with sepsis-related ARDS indicated that administration of ~ 15 g/day of IV vitamin C for 4 days may decrease mortality in these patients [3]. An earlier IV vitamin C trial of patients admitted to the ICU with pneumonia included hydrocortisone administration [4], however, systemic corticosteroid treatment has not been shown to have significant benefits in patients with COVID-19 [5].

Just recently registered on clincialtrials.gov (Identifier: NCT04264533), a new clinical trial to investigate vitamin C infusion for the treatment of severe 2019-nCoV infected pneumonia has begun in Wuhan, China. This is one of the first RCTs to test the effects of IV vitamin C in patients infected with this virus. In this trial, the investigators will treat 140 patients with a placebo control or intravenous vitamin C at a dose of 24 g/day for 7 days. They will assess requirements for mechanical ventilation and vasopressor drugs, organ failure scores, ICU length of stay and 28-day mortality.

The investigators of the new study hope to complete the trial by the end of September. Although the findings of this trial will be too late for the many thousands of people currently infected with the virus, the study will nevertheless provide valuable information as to the potential mitigation of symptoms by vitamin C during future viral outbreaks.

## Data Availability

N/A
